# Rapid Isolation and Concentration of Pathogenic Fungi Using Inertial Focusing on a Chip-Based Platform

**DOI:** 10.3389/fcimb.2019.00027

**Published:** 2019-02-12

**Authors:** Beth Burgwyn Fuchs, Soraya Eatemadpour, Joseph M. Martel-Foley, Shannon Stott, Mehmet Toner, Eleftherios Mylonakis

**Affiliations:** ^1^Rhode Island Hospital, Alpert Medical School and Brown University, Providence, RI, United States; ^2^BioMEMS Resource Center, Center for Engineering in Medicine and Surgical Services, Massachusetts General Hospital and Harvard Medical School, Boston, MA, United States; ^3^The Center for Engineering in Medicine, Massachusetts General Hospital and Harvard Medical School, Boston, MA, United States

**Keywords:** *Candida*, cell separation, fungal diagnostics, fungi, IFF, inertial focusing, microfluidics

## Abstract

Systemic *Candida* infections remain a leading cause of nosocomial infections in the United States and worldwide. Many challenges remain in achieving rapid, direct diagnosis of fungal bloodstream infections due to limitations of conventional diagnostic methods that continue to demonstrate poor sensitivity, prolonged culture times that lead to delayed treatment, and detection variability between tests that compromises result reproducibility. Despite advancements in technology, mortality, and cost of care presented by blood stream infection with *Candida* spp. (candidemia) continues to rise and there is an urgent need for the development of novel methods to accurately detect *Candida* species present within the blood. This is especially true when patients are infected with drug resistant strains of *Candida* where accurate and immediate therapeutic treatment is of the importance. This study presents a method of separating fungal cells from lysed blood using inertial forces applied through microfluidics in order to abbreviate the time required to achieve a diagnosis by mitigating the need to grow blood cultures. We found that *C. albicans* can segregate into a focused stream distinct from white blood cells isolated within the Inertial Fungal Focuser (IFF) after red blood cell lysis. As a result of the focusing process, the collected cells are also concentrated 2.86 times. The same IFF device is applicable to non-*albicans* species: *Candida parapsilosis, Candida glabrata*, and *Candida tropicalis*, providing both isolation from lysed blood and a reduction in solution volume. Thus, the devised platform provides a means to isolate medically significant fungal cells from blood and concentrate the cells for further interrogation.

## Introduction

Life-threatening fungemias can result from the introduction of *Candida* spp. or other fungal organisms to the bloodstream, with the potential to become a debilitating disease to both community and hospital-based populations. Compared to localized infections, systemic fungal infections often present higher patient mortality, lengthened hospital stays, and burdening healthcare costs (Zaoutis et al., 2010).

Since bacterial infections are more frequent than fungal infections, empiric antimicrobial treatment is often directed to these microbes, and usually includes at least one antimicrobial targeted against *Staphylococcus aureus*, along with at least one antibiotic against gram-negative bacteria (Micek et al., 2005). Before positive fungal blood culture results, antifungals are only added to the regimen at the physician's discretion and usually only recommended for high-risk patients (Morrell et al., 2005). A retrospective cohort analysis conducted by Morrell et al. in 2005 found that patients who received antifungal treatment within the first 12 h of infection had a lower risk of hospital mortality than patients who were administered treatment after 12 h (Morrell et al., 2005), thus showing the need for rapid diagnosis.

The most prominent fungal infection is elicited by *Candida albicans*. However, a larger number of non-*albicans* related bloodstream infections are also problematic. In the United States, *Candida* species rank among the top five opportunistic pathogens for nosocomial infections, with *C. albicans, Candida glabrata, Candida parapsilosis, Candida tropicalis*, and *Candida krusei* responsible for 95% of all occurring infections (Wisplinghoff et al., 2004; Pappas, 2006; Pfaller and Diekema, 2007; Sievert et al., 2013; Yapar, 2014). The non-*albicans* species are often seen in patients with cancer or hematological malignancies, and exhibit a level of virulence that results in significant mortality (Krcmery and Barnes, 2002).

Numerous systems have been proposed to detect microbial pathogens from blood to improve the diagnostic process for systemic infections. Conventional tests include manual and semi-automated methods that are based on morphological and physiological characterization (Goodwin et al., 1992). Blood culture analysis followed by antimicrobial susceptibility testing is the most common approach for identifying systemic bacteremias and fungemias (Pardo et al., 2014). However, many concerns have pointed to limitations in the sensitivity, reliability, and timeliness of automated blood culture methods. While newer technologies and commercially available kits have been developed to better meet the challenge of an earlier diagnosis, blood culture remains the standard, and most frequently used methods of detection (Morris et al., 1995; Vitale and Nucci, 2014).

A test of the BacT/Alert automated blood culture system, the current gold standard device for fungal diagnostics, found that *Candida* growth was detected in 74% (479/648) of seeded culture bottles, indicating that infections can be missed based on growth (Horvath et al., 2007). The sensitivity of the assay decreases with low inoculum concentration [1,000 yeast cells/bottle (79%); 10 yeast cells/bottle (70%)], with a mean detection time of *C. albicans* at 20–30 h, depending on bottle type and inoculum concentration (Horvath et al., 2007).

In this report we utilize the differential morphological elements of fungal cells in order to separate *Candida* cells from lysed blood using microfluidics. The isolation process entails lysing red blood cells (RBCs) then flowing the fluid through an inertial microfluidic device to separate fungal cells from white blood cells (WBCs), generating a concentrated fungal specimen.

We applied the described dynamic inertial forces afforded through microfluidics in order to separate fungal cells, particularly *Candida* species, from lysed blood, generating a concentrated sampling that contains many of the contaminating agents. In this report, we present our findings and describe the efficacy in the separation process and recovery of the fungal cells. This technique holds the potential to be a valuable compliment to automated diagnostics systems by mitigating the needs for prior culturing or centrifugation methods.

## Materials and Methods

### Master Molds Were Produced Using SU-8 Photolithography

The molds were created following the protocol described by Martel and Toner (2012). To create the master mold, a silicon wafer was dehydrated at 200°C for 20 min before being exposed to a high power oxygen plasma to promote adhesion. The SU8-50 epoxy-based negative photoresist (Microchem Corp, Westborough, Massachusetts) was spun at 2,650 rpm to achieve a thickness of 50 μm. Two baking periods occurred at 65°C for 10 min and 100°C for 20 min, respectively. After cooling, SU8-50 was exposed to UV light through a mylar emulsion printed photomask with channel features designed to split flow before two more baking periods at 65°C for 10 min and 100°C for 20 min. The master silicon wafer was developed in the BTS-220 SU8-Developer (J.T. Baker, New Jersey) and measured via profilometry to verify the dimensions of raised SU8 features.

### Fabrication of PDMS Microchannels

A 10:1 mixture of Sylgard 184 elastomer base and curing agent (Dow Corning, Midland, Michigan) was prepared and poured on top of the silicon wafer and submitted to vacuum desiccation to remove any bubbles. To cure the PDMS, the mold was incubated at 65°C for at least 8 h. The cured PDMS was then cut along the edge of the wafer to remove the slab that contained the microchannel design and was diced into individual channels or in groups of two and sharpened needle tips were inserted to establish the locations of both the inlet and outlet. To remove remaining particulate, the devices were then cleaned with low-residue tape and oxygen plasma and activated PDMS bonded to 1 mm thick glass microscope slides. The bonded devices were baked at 75°C for 10 min and tubing was press fit and glued to the inlet and outlet locations using Loctite Medical Grade Epoxy 4013 with consideration to maintaining flow capability. The developed device was designated the Inertial Fungal Focuser (IFF).

### Strains and Culture Conditions

Fungal reference strains used in the described studies are listed in Table 1. The strains were stored at −80°C until needed. Fungi were grown in yeast extract, peptone, and dextrose (YPD) media with agitation at 30°C unless otherwise stated.

**Table 1:** Microbial strains used in this study.

**Fungi**	**Strain**
*Candida albicans*	SC5314 (CAN14)
*Candida glabrata*	ATCC 90030
*Candida parapsilosis*	ATCC 22019
*Candida tropicalis*	ATCC 13803

### Cell Staining

Fungal cells were stained with Fluorescein isothiocyanate (FITC) to observe movement as they passed through the IFF. An overnight culture of *C. albicans* strain CAN14 cells was washed twice with phosphate buffered saline (PBS) and collected with centrifugation. Cells were counted with a hemocytometer and suspended at a concentration of 10^6^ cells/mL. FITC (10 mg/mL in DMSO) was added to achieve a final concentration of 0.1 mg/mL in PBS. Cells were incubated for 30 min at room temperature in the dark. Subsequently, cells were washed three times in PBS containing 10 mg/mL bovine serum albumin (BSA). Cells were then subjected to focusing with the IFF.

WBCs were also stained to better document their movement through the IFF. WBCs isolated from whole blood using a standard ammonium chloride lysis procedure were stained using 1M Calcein AM red/orange (ThermoFisher Scientific, Waltham, Massachusetts) stock solution at a ratio of 1 uL of stain for each 1 mL of cell suspension, then mixed constantly for 15 min. The suspension was washed using centrifugation at 50 g for 5 min with 1X PBS containing 2% fetal bovine serum (FBS) and resuspended in the same buffer to the operating cell concentration based upon the whole blood CBC measurement (1 × 10^6^ cells/mL).

### Migration of Fungal Cells Suspended in PBS Through the IFF Device

A culture of *C. albicans* reference strain CAN14 was grown overnight with agitation at 30°C. Cells were collected and washed with PBS and stained with FITC as described above. Cells were then enumerated with the aid of a hemocytometer and suspended at a concentration of 1,600 cells/mL in PBS. A 20 mL aliquot of cells were filtered (Millex-SV 5.0 μm filters, ThermoFisher Scientific, Waltham, Massachusetts) and prepared for the IFF devices. Devices were primed with 5 mL of 0.01% sodium dodecyl sulfate (SDS) solution at a flow rate of 400 μL/min using a Harvard Apparatus syringe pump before 5 mL of cells suspended in PBS were run through the devices. Material isolated from each of the three exit ports was collected and 100 μL from each port was plated onto YPD containing 45 μg/mL kanamycin, 100 μg/mL ampicillin, and 100 μg/mL streptomycin. YPD plates were incubated at 30°C for 2 days before colony forming units (CFU) were counted.

### RBC Lysis

10X RBC lysis solution (Miltenyi Biotec, Auburn, California) was diluted to 1X with ddH_2_O. Serial dilutions of CAN14 cells exposed to 1X RBC lysis solution between 0 and 24 h was spotted on YPD media and grown at 30°C for 2 days to observe growth.

### Fungal Cell Migration Through IFF Device Using Lysed Blood

A culture of *Candida* cells was grown overnight in YPD with agitation at 30°C. Cells were collected through centrifugation and washed twice with PBS. They were then enumerated with a hemocytometer and suspended at a concentration of 1,600 cells/mL in whole blood. The 5 mL spiked blood sample was added to 45 mL of 1X Red Blood Cell lysis buffer (Miltenyi Biotech, Inc., Auburn, CA). To remove debris created during hemolysis, the blood-buffer solution was passed through a 5.0 μm filter (Millex, Millipore Sigma, St. Louis, Missouri) to remove coarse particles that could clog the IFF. Devices were primed with 5 mL of 0.01% SDS at a flow rate of 400 μL/min using a Harvard Apparatus syringe pump before the blood samples were tested. The blood-buffer solution was then run through the IFF at a flow rate of 400 μL/min, collecting material in three designed exit ports. To determine the direction of fungal cells, 100 μL from each port was plated onto YPD containing 45 μg/mL kanamycin, 100 μg/mL ampicillin, and 100 μg/mL streptomycin. Plates were incubated at 30°C for 2 days before CFUs were counted.

### Identification of Isolated *C. albicans*

To determine whether fungal DNA can be amplified from blood submitted through the IFF device without prior fungal DNA extraction, PCR was carried out to analyze samples of IFF-processed material using the Hemo Klen Taq Kit (New England Biolabs, Ipswich Massachusetts). For the 20 μL reaction mix, 1 μL of IFF-processed sample was mixed with 19 μL of master mix containing 1X PCR buffer, 62.5 μM dNTPs, 0.375 μM of AF114470 forward (5′-GGGAGGTAGTGACAATAAATAAC-3′) and reverse (5′- CGTCCCTATTAATCATTACGAT-3′) primers (Jaeger et al., 2000), Hemo Klen taq polymerase, and sterile water. Thermal cycler parameters comprised of heating the samples at 95°C for 3 min followed by 35 cycles of 95°C for 20 s, 50°C for 2 min, and 68°C for 2 min. A final extension at 68°C was applied for 10 min.

The amplified PCR product was cloned into a pGEM T-easy vector (Promega, Madison, WI). The generated plasmid containing the insert was sequenced to confirm the 18S rRNA (Genewiz, South Plainfield, NJ).

## Results

Cell manipulation using microfluidic inertial forces allows for different cell types to be focused into unique fluidic equilibrium positions that are determined by biophysical characteristics of the different cell types. Chiefly, the cell size and shape influence movement in a microfluidic channel; the equilibrium positions are further impacted by the channel curvature and fluid speed. Fungal cells in the planktonic form differ in size between species but are, on average, smaller than WBCs that range from 7 to 25 μm between the various types of leukocytes. The majority of WBCs are contributed by neutrophils that comprise 60 to 75% of the leukocytes within the blood and on average are 12 to 15 μm. *Candida* cells range in maximum diameter from 2 to 11 microns. Planktonic *Candida* cells are ellipsoidal or spherical, compatible with automated methods to isolate the fungi from other cells present in the blood.

### Microfluidic Device Design

**Figure 1:**
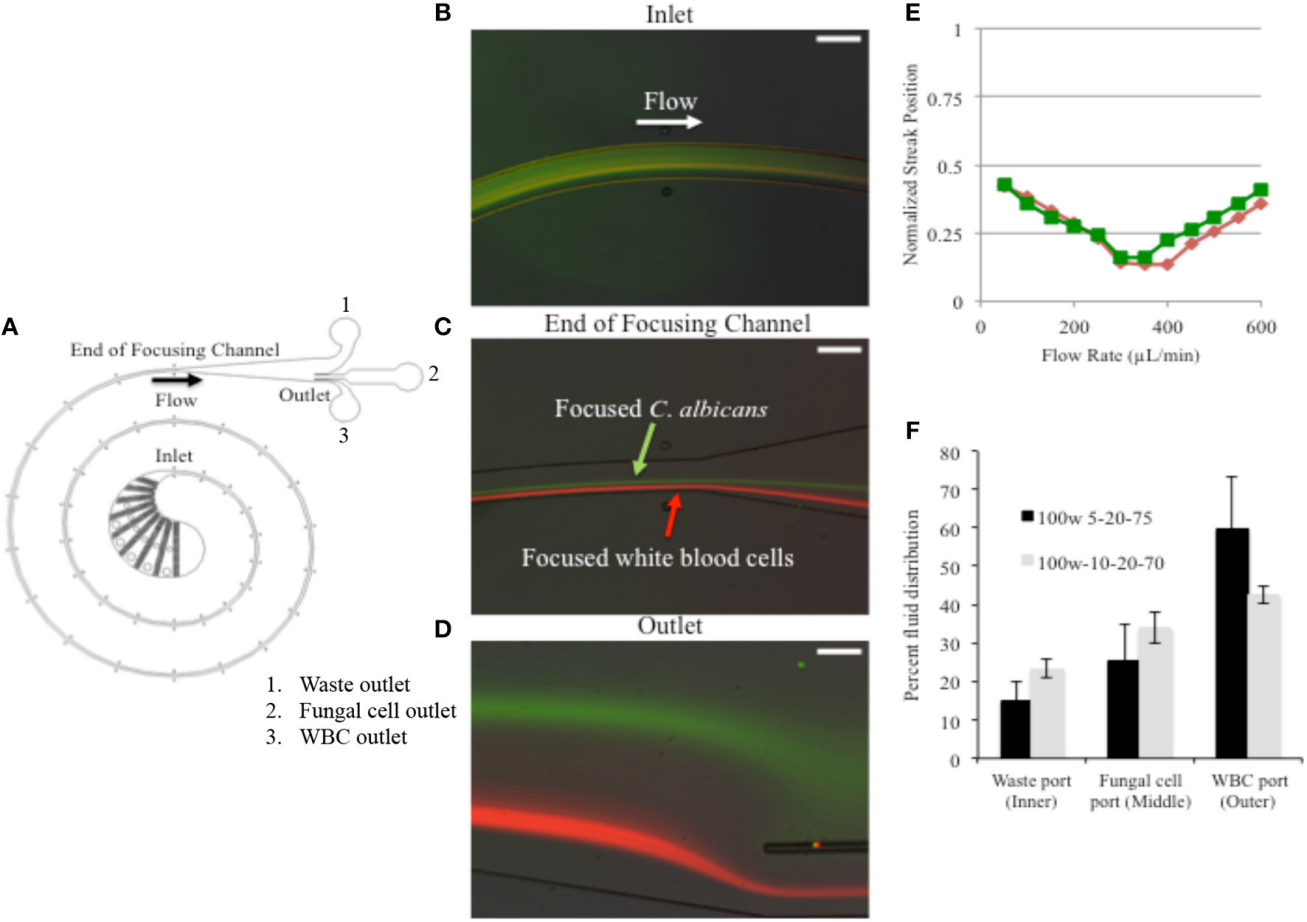
Fungal cells were FITC stained to differentiate from calcein stained WBCs. **(A)** Device schematic. **(B)** A mixture of cells that includes stained WBCs and fungal cells enters the IFF without prior separation and are subjected to the microfluidic forces and specified flow rate. **(C)** As cells travel through the path of the coil, at a specific flow rate of 400 μL/min, *C. albicans* are focused into a stream. **(D)** By the time they reach the outlet, the cell form two distinct streams. **(E)** The lateral separation of cell types was tested over a range to detect the optimal applied flow rate. **(F)** The distribution observed at device 100w-5-20-75 is shown in black and distribution for IFF device design 100w-10-20-70 is shown in gray. Error bars indicated standard deviation.

A device design template based upon the size of white blood cells was utilized for the spiral inertial focusing channel which was then edited to incorporate a larger filter section (Martel and Toner, 2012). The outlet design was tailored to the results of initial testing showing the focusing locations of WBCs and fungal cells at different flow rates. The final design is shown in Figure 1A. The channel is 100 μm wide, 50 μm tall, and ~4 cm long. The fundamentals of inertial focusing are described elsewhere, but briefly, due to high fluid velocities in the microchannel hydrodynamic forces on the cells cause them to move to specific cross-sectional locations in the channel dependent upon channel design, flow parameters, and cell characteristics.

### Flow Rate

We were able to identify 400 μL/min as the optimal flow rate to separate fungal cells from WBCs in PBS suspension. Fungal cells were stained with FITC, enabling real time observation while moving in the IFF device. The fungal cells were observed to enter the device with a random distribution, and then focused into an equilibrium position within the channel during the flow (Figures 1B–D). At this same flow rate, FITC stained fungal cells were found to separate from WBC (stained with calcein orange) to form separate focused streams along the channel (Figures 1C,D), thus showing that *C. albicans* could be focused and separated from WBCs. The flow rate was tested over a range of 50 to 600 μL/min. Using fluorescent images to locate the streams of WBCs and fungal cells the lateral separation distance was found to be maximized at a flow rate of 400 μL/min. The separation distance is highlighted in Figure 1E, where the positions of the fluorescent peaks are plotted vs. flow rate.

### *C. albicans* Cells Are Concentrated With Passage Through the IFF

We tested two different PDMS microchannel outlet designs to determine the best means of fluid distribution with the goal of reducing the final fungal cell suspension volume after IFF processing, thus concentrating the fungal material. The two device designs are distinguished by the relative hydraulic resistances of the three outlets intended to split the flow into the different collection ports. Device 100w-5-20-75 was designed to direct 5% of liquid to an inner outlet intended for waste, 20% of liquid into a middle outlet intended for isolated fungal cells, and 75% of liquid to an outer outlet that should receive WBCs. Alternatively, device 100w-10-20-70 was designed to split 10, 20, and 70% of liquid into the respective outlets. Therefore, the final volume directed to the middle outlet (the fungal collection outlet) would be reduced from a 5 mL starting sample to ~1 mL.

We found discrepancies between the designed and the observed ratios (Figure 1F). Approximately 40–60% of liquid was directed to the outer outlet for both types of tested devices, while 15–25% of liquid was directed to the inner outlet. However, the amount of liquid directed to the middle outlet stayed between 25 and 34% ± 4–9.1%, which matches the design ratios more closely and confirms that a 5 mL starting volume was consistently reduced to a final volume of 1.3–1.7 mL at the middle outlet in an effort to concentrate the fungal cells. Of the two designs, 100W-5-20-75 showed greater volume reduction and was favored for further examination.

### Non-*albicans* Species of *Candida* Are Concentrated With the IFF

We sought to determine the extent to which the IFF device is able to direct fungal cells to the appropriate collection outlet (middle port) and look at design conservation by testing several non-*albicans* species to determine if the IFF could be used for fungal cells more generally. In evaluating the separation proficiency of several species of *Candida*, the distribution of colony forming units was expected to present some variability due to differences in size between species. Table 2 provides the percentage of CFU obtained from each port throughout a series of trials that interrogate 10-fold serial dilutions of non-*albicans Candida* subjected to IFF. Starting 5 mL PBS samples spiked with fungal cells at concentrations of 1,600, 160, and 16 cells/mL resulted in the majority of fungal cells being directed to the appropriate middle outlet. For *C. albicans*, over 80% of the recovered fungal cells in the 8,000, 800, and 80 cell concentrations per 5 mL were correctly directed. However, the number of CFUs indicates that there was fungal cell loss throughout the trial. A portion of the cells from all of the tested *Candida* species (*C*. *albicans, C*. *glabrata, C*. *parapsilosis*, and *C*. *tropicalis*) was effectively directed to the fungal collection port. Among the recovered cells, *C. albicans* was most efficiently directed to the appropriate collection location. Using the same IFF design, direction toward the middle port of *C*. *parapsilosis* fell between 69 and 74% among the captured cells, proving to be the second most amenable to the separation procedure.

**Table 2:** Percentage of recovered *Candida* species cells distributed among collection ports when using PBS as a flow medium.

		**Outer**	**Middle**	**Inner**
1,600 cells/mL	*C. albicans*	8.3 ± 8.7	90.0 ± 8.0	1.67 ± 2.0
	*C. glabrata*	25.4 ± 4.7	61.7 ± 6.0	12.9 ± 1.4
	*C. parapsilosis*	5.3 ± 4.8	73.6 ± 4.6	21.1 ± 0.5
	*C. tropicalis*	41.4 ± 1.6	52.9 ± 1.6	5.70 ± 3.2
160 cells/mL	*C. albicans*	11.2 ± 22.4	85.6 ± 21.2	3.2 ± 6.3
	*C. glabrata*	34.7 ± 7.8	55.9 ± 6.6	9.4 ± 2.6
	*C. parapsilosis*	5.7 ± 9.9	69.7 ± 10.7	24.6 ± 15.9
	*C. tropicalis*	33.2 ± 13.6	62.6 ± 19.6	4.2 ± 6.0
16 cells/mL	*C. albicans*	2.1 ± 4.2	91.2 ± 10.3	6.8 ± 8.9
	*C. glabrata*	13.3 ± 5.4	67.6 ± 21.9	19.0 ± 16.5
	*C. parapsilosis*	7.3 ± 8.5	74.1 ± 9.1	18.6 ± 9.2
	*C. tropicalis*	8.1 ± 11.4	85.7 ± 2.6	6.3 ± 8.8

For *C. glabrata*, the percentage of cells that were directed to the middle outlet is lower, with only 55–70% correct fungal cell migration. When tested with starting samples at greater cell densities, a large percentage of *C. glabrata* fungal cells flow to the bottom outlet intended for WBCs. This may be due to the fact that *C. glabrata* is considerably smaller with a spherical shape compared to the other *Candida* species and current design aspects may not be optimal for higher degrees of isolation.

**Figure 2:**
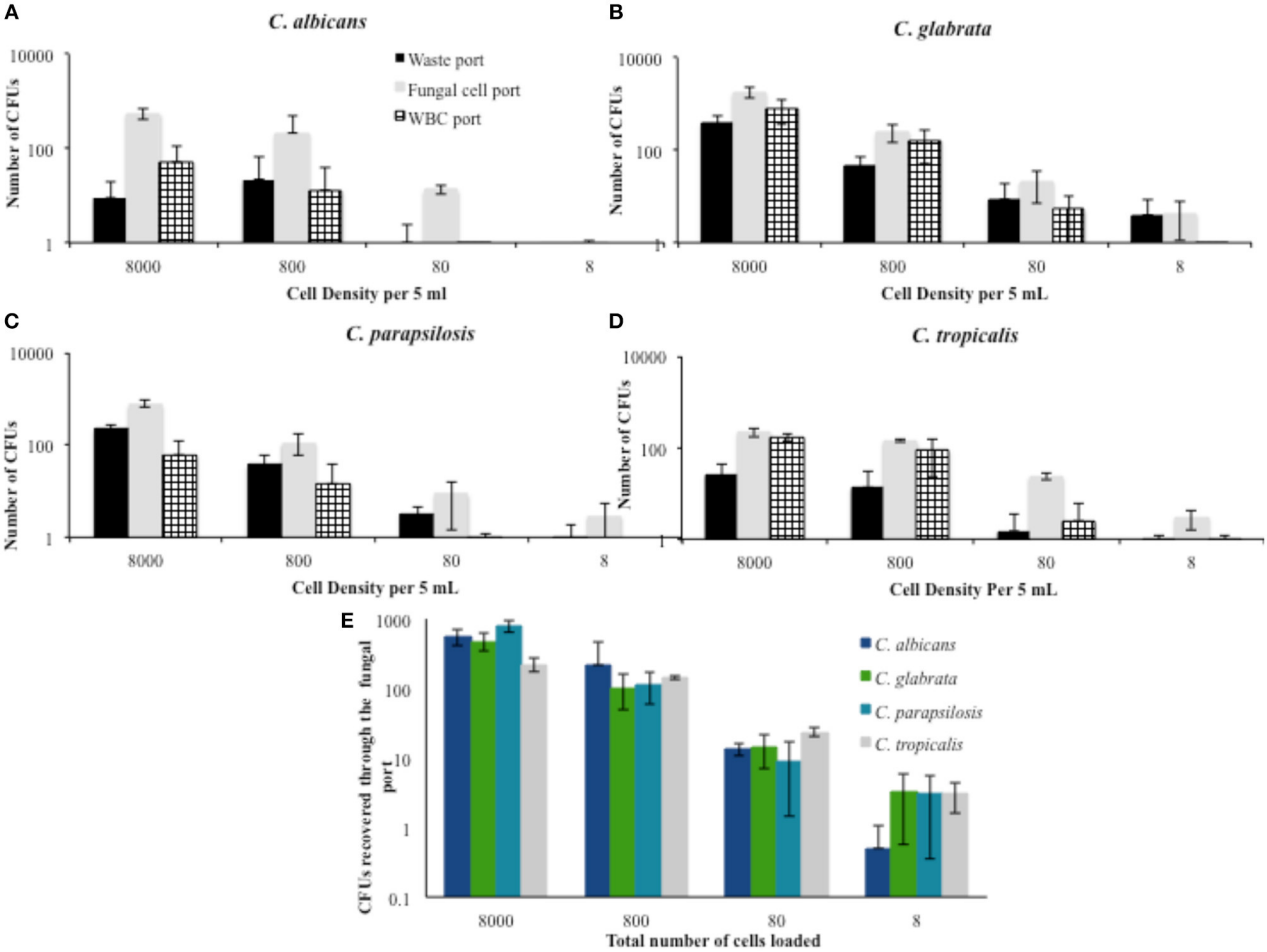
Percent distribution of fungal cell migration at different cell densities in PBS solution. The portion of fungal cells directed to the waste port, fungal cell port, and WBC port were determined for the most commonly encountered fungal species. **(A)** *C. albicans* **(B)** *C. glabrata* **(C)** *C. parapsilosis*, and **(D)** *C. tropicalis*. **(E)** A summary of the recovered cells from the fungal cells port is provided as a comparison between the tested *Candida* spp. Error bars indicated standard deviation.

Apart from observing the percentage of cellular distribution at these concentrations, the number of CFU recovered from each device was also assessed. Figure 2 shows the corresponding number of CFUs for each concentration during the trials conducted in PBS. The number of viable cells for all species was considerably lower than the amount spiked in the starting sample. For the 8,000 cells per 5 mL density, 5–10% of *C. albicans* cells were recovered from the different trials. Although there was variability seen in the number of CFUs across different cell densities, recovery appeared to improve as the density of the sample decreased. At a spiked concentration of 800 cells per 5 mLs, an average of 26.5% of the fungal cells were recovered.

### RBC Removal

For the interrogation of fungal cell spiked blood in the IFF, RBCs must first be depleted from solution. To this end, mammalian cells must be lysed without adversely affecting the fungal cells. To accomplish RBC removal we take advantage of the differing cell structures between the cell types; fungi have a cell wall that protects against stress and damage, a structure that serves as an asset environmentally, and is utilized in our processing methodology. *C. albicans* were exposed to 1X RBC lysis solution for up to 24 h, diluted, and plated on yeast media to see if the lysis solution killed the fungi or retarded growth. We found that incubation for 24 h in 1X RBC lysis solution had no effect on the growth of *C. albicans* (Supplemental Figure 1). Thus, the 1X RBC lysis solution could be used on blood cultures containing fungal cells to lyse the RBCs without reducing the population of *C. albicans* within the specimen.

### *C. albicans* Cells Are Isolated From Lysed Blood With the IFF

Once segregation of *C. albicans* was achieved in PBS solution, the performance of the IFF device in concentrating fungal cells from blood was investigated. Whole blood samples were obtained at Rhode Island Hospital (after review from the Rhode Island Institutional Review Board where the study was determined to be exempt) and were treated with anticoagulant, kept on a rotator, and 5 mL aliquots of blood were spiked with *C. albicans* concentrations of 1,600 cells/mL. RBC lysis was performed quickly in order to prevent clot formation that would affect flow rate when submitted through the IFF device. The 5 mL fungal spiked blood was diluted 10:1 (RBC lysis buffer: blood) and filtered before subjection to IFF.

**Figure 3:**
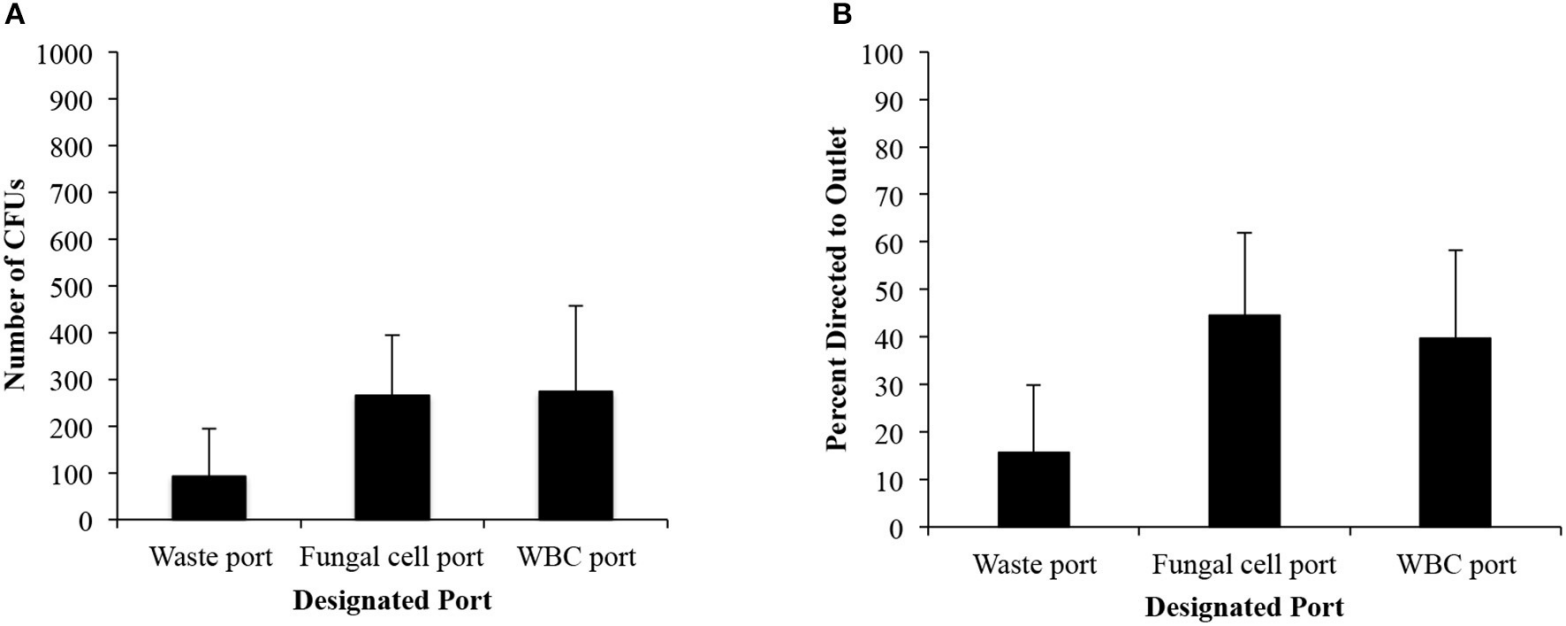
IFF data for *C. albicans* in lysed blood. **(A)** Distribution of the raw number of CFUs. **(B)** Percent distribution of *C. albicans* CFU between each port from cells spiked in whole blood from a starting concentration of cells was 8,000 cells per 5 mL sample. Error bars indicated standard deviation.

Figure 3A illustrates the number of colony forming units and the distribution of fungi to each port. While the number of colony forming units confirms that the majority of cells were directed to the middle outlet, the percentage of distribution was <50% (44.6% for the 5-20-75 IFF device), lower than the percentage seen from *C. albicans* suspensions in PBS (Figure 3B). The number of viable cells that were recovered from the sample was similar between the fungal collection port and the bottom port intended for WBCs. However, the waste port continued to have the lowest number of CFUs. We recovered 265 ± 137 CFUs among the inoculated 8,000 CFUs in the middle port for the 5-20-75 IFF device. Among all the ports, we achieved a total recovery rate of 8.4% of the fungal cells that were spiked into the blood. The viscosity of the blood and the presence of RBC remnants are potential factors to account for the difference in fungal cell isolation efficiency between spiked whole blood vs. PBS samples using the 5-20-75 IFF device.

### Confirmation of Recovered Cells

To validate the capabilities of the IFF device, measures were taken to ensure that the cells isolated from blood were indeed *C. albicans* and not from other contaminants that may be present within the blood. To verify that the PCR reaction was correctly identifying the presence of *C. albicans* from the fluid collected from the fungal collection port of the IFF device, the PCR products were sequenced. An aliquot of the collected material was subjected to PCR directly, and the amplified product (Supplemental Figure 2) was sequenced for identification. These findings suggested that *Candida* could be detected from blood and the fungal cells concentrated and subjected to further identification analysis to aid in improved diagnostics.

## Discussion

Early diagnosis and timely initiation of appropriate antifungal therapy is critical in order to address the significant morbidity and mortality associated with invasive fungal infections, and especially candidemia (Kourkoumpetis et al., 2012; Arvanitis et al., 2014). Here we present a means of isolating and concentrating fungal cells from biological fluid. The intention is to mitigate the need for culturing, a time consuming step. The presented IFF device and methodology also shows that cells can be concentrated into small volumes which stands to complement new technologies being devised for the next generation of fungal diagnostics devices which focus on molecular approaches.

Our platform utilizes the phenomenon of inertial focusing to isolate fungal contaminants from lysed blood remnants. Inertial focusing occurs at finite Reynolds number, when all objects passing through microchannels experience both wall interaction, and shear gradient lift forces. The fundamental assumption behind the wall interaction force is that unless a particle is small enough to act as a fluid parcel, it will interact with the walls of a microchannel causing the particle to move slightly slower than the fluid molecules as well as a buildup of pressure between the wall and the particle. This generates a force directed away from the channel's walls. This then becomes coupled to Shear gradient lift force when a particle is experiencing unequal shear forces due to the parabolic nature of the fluid velocity profile in the microchannel, generating a force toward the walls of the channel where this unequal stress is minimized.

Additional forces can be applied to aid in the separation of component within the fluid using channel design elements. For example, curved channels promote lateral motion through introduction of a secondary flow and resulting drag force perpendicular to the main flow, thus altering the equilibrium positions (Martel and Toner, 2012; Nivedita et al., 2017). Spiral channels maintain this secondary flow, called Dean flow, over the entire channel length creating a more stable equilibrium position location as compared to asymmetrically curved channel designs (Gossett and Di Carlo, 2009). The ability to change the curvature of spiral microchannels holds opportunity for better control and further optimization of flow rates amenable for high-throughput particle separation (Kuntaegowdanahalli et al., 2009).

The application of these fluid forces through microfluidics is regularly explored as a means of separating cells and other bioparticles based upon size (Martel and Toner, 2014). One of the most common applications is separating circulating tumor cells (CTCs) from blood cells due to the tendency for CTCs to be larger. Of significance is that CTCs can appear in the blood stream during metastasis at a similar rate to fungal cells during candidemias (<100/mL of whole blood). While smaller cells are more problematic to focus due to the nature of the forces scaling strongly with size, fungal cells have been focused previously in straight channels (Masaeli et al., 2012) and thoughtful designs have utilized the size of blood cells to focus RBC in an effort to separate from bacteria cells (Mach and di Carlo, 2010). The high throughput nature of inertial focusing is necessary for dealing with low concentration targets, and makes it a promising technique for isolating fungal cells.

Isolating microorganisms from blood differs from the current diagnostic convention of growing the microbes from a blood culture. The goal of both is to recognize the presence of microbes. Culturing relies on microbe growth in order to become recognizable. A positive blood culture test result non-specifically indicates the presence of bacteremia and/or fungemia, so additional time and more specific methods are required. This time consuming process can be greatly reduced by isolating fungi directly from blood rather than waiting for growth. Furthermore, rapid isolation can significantly impact patients infected with drug resistant strains of *Candida* or non-*albicans* species that are less susceptible to commonly provided antifungal therapeutics.

Culture methods are lengthy, requiring as much as 5-day incubation period to ensure adequate time for microbial growth. While studies have found that automated blood culture methods indicate positive microbial growth within the first 48 h with a median time of 12–17 h, the time frame for appropriate antimicrobial intervention is <6 h after the onset of symptoms (Jordana-Lluch et al., 2013; Pardo et al., 2014). Indeed, at a flow rate of 400 μL/min fungal cells are separated from lysed whole blood samples within 125 min using the IFF, thus available for further identification methods such as PCR.

Others have shown that *C. albicans* can be isolated from the blood using alternate or augmented microfluidic methodologies. Cooper et al. found that 67% of seeded fungi in blood can be separated and optically visualized using ligand coated beads to capture and isolate the cells with magnetic force (Cooper et al., 2014). A major difference when compared to the current report is that the IFF does not require magnetic manipulation to isolate rare cells as developed by Cooper et al. which requires 1,000-fold excess of magnetics beads relative to pathogen. Instead, we take advantage of size differentials that become amplified in spiral microchannels compared to lateral flow applied in the separation procedure by Cooper et al.

Javanmared et al. report capturing 6.4% of the total *C. albicans* cells that pass through their microfluidic device (Javanmard et al., 2012). The platform is based on parallel formatted channels coated with fungal specific antibodies. By binding the pathogen cells to the wall of the channel, they create an imaging opportunity that can aid with identifying the pathogen when an infectious state occurs.

Two areas in which microfluidic processing has enabled efficiency are volume reduction and sample concentration as an alternative to traditional centrifugation (Martel et al., 2015). Biological and clinical assays traditionally accomplish volume reduction through centrifugation in order to purify and enrich a sample for further analysis. Continuous flow microfluidic devices not only offer increased sample concentration and volume reduction, but also avoid the potential to damage cells, and alter cell gene expression that may occur when bioparticles are exposed to high centrifugal forces (Soto et al., 2007; Peterson et al., 2012). Furthermore, such devices do not require that a large starting volume be split into smaller samples in order to be processed, which increases the risks of contaminating and/or losing the sample (Martel and Toner, 2014).

When considering influencing factors, such as the size of inoculum upon detection of *Candida* bloodstream infections, time presents an important barrier for efficient detection, and treatment. In the BACTEC system, inoculum size heavily influences growth detection and time to detection for *Candida* BSIs, where larger inocula lead to faster detection and smaller inocula delay detection (Jacoby, 2005). Using the current gold standard, the BACTEC blood culture system results are slow and plagued with challenges such as poor detection in anaerobic media as well as false results even when testing large inocula of 100 CFU/mL. This is especially a disadvantage when trying to detect growth in patients with candidemia, where the circulating fungal cells concentration is <10 CFU/mL. This low contaminate concentration could be well-addressed by the use of microfluidic processing. The isolation of each *Candida* spp. could then be determined based upon morphological characteristics that distinguish the fungal cells from mammalian cells.

In conclusion, we postulate that concentrating fungal cells directly from clinical specimen can aid the emergence of a more rapid diagnostic platform. Even with this collection of differences among the fungal population, the design of the IFF device has proven it is able to achieve a focused stream of fungal cells that can be directed for isolation. Although the IFF does not segregate 100% of the *Candida* cells it is capable of volume reduction and solute concentration to capture fungal cells present in small quantities at a rapid rate. The IFF technology could be incorporated with other diagnostic advances that mitigate the need for centrifugation or incubation of the starting material. While the device does not directly quantify cells, it does provide a means to qualitatively detect the presence of fungal cells quickly, a potentially valuable piece of information for clinicians, holding the potential to aid in early diagnosis.

## Author Contributions

Design was provided by BF, JM-F, SS, and MT. Device testing was accomplished by BF, JM-F, SS, and SE. Data analysis was performed by BF, JM-F, SE, MT, and EM. SE and BF wrote the first draft of the manuscript and all authors contributed to the final manuscript preparation.

### Conflict of Interest Statement

The authors declare that the research was conducted in the absence of any commercial or financial relationships that could be construed as a potential conflict of interest.

## Supplementary Material

Supplemental Figure 1 Fungal resistance to lysis. *C. albicans* cells incubated in 1X RBC for 24 h and serial diluted before plating on YPD show no signs of growth inhibition after exposure to the lysis buffer.

Supplemental Figure 2 PCR samples tested from collected material of blood submitted through IFF device. Lane 1 provides a 100 bp ladder for reference. Lane 2 is the product obtained from colony PCR using 1 μL of extract collected from IFF device with 19 μL of master mix.
