# Supplementary figures and images for: Rapid Isolation and Concentration of Pathogenic Fungi Using Inertial Focusing on a Chip-Based Platform
Source: Front Cell Infect Microbiol. 2019 Feb 12;9:27. doi: 10.3389/fcimb.2019.00027 (PMC6379272; doi:10.3389/fcimb.2019.00027)

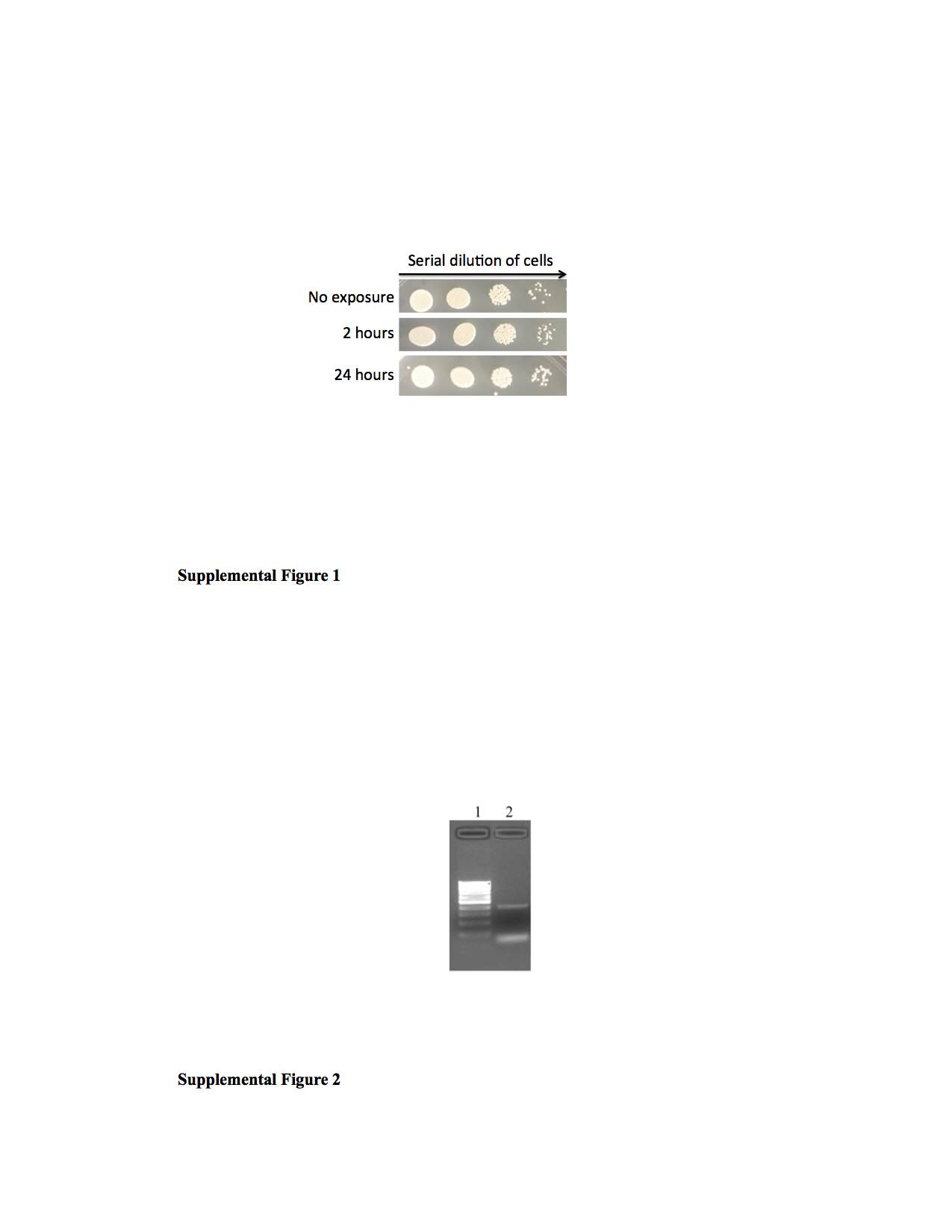

Supplement: Supplemental Figure 1 — Fungal resistance to lysis. C. albicans cells incubated in 1X RBC for 24 h and serial diluted before plating on YPD show no signs of growth inhibition after exposure to the lysis buffer. [file Image_1.jpg]
